# Potential role of exercise-induced glucose-6-phosphate isomerase in skeletal muscle function

**DOI:** 10.20463/jenb.2019.0014

**Published:** 2019-06-30

**Authors:** Seong Eun Kwak, Hyung Eun Shin, Di Di Zhang, Jihyun Lee, Kyung Jin Yoon, Jun Hyun Bae, Hyo Youl Moon, Wook Song

**Affiliations:** 1 Institute of Sport Science, Seoul National University, Seoul Republic of Korea; 2 Institute on Aging, Seoul National University, Seoul Republic of Korea

**Keywords:** Aging, Muscle function, Glucose-6-phosphate isomerase, C2C12 cell, Skeletal muscle

## Abstract

**[Purpose]:**

Recent studies have shown that glucose-6-phosphate isomerase (GPI)—which is a glycolysis interconversion enzyme—reduces oxidative stress. However, these studies are limited to tumors such as fibrosarcoma, and there are no studies that have examined the effects of exercise on GPI expression in mice skeletal muscle. Furthermore, GPI acts in an autocrine manner thorough its receptor, autocrine motility factor receptor (AMFR); therefore, we investigated expression level changes of secreted GPI from skeletal muscle in *in vitro* study to examine the potential role of GPI on skeletal muscle.

**[Methods]:**

First, we performed an in vitro study, to identify the condition that upregulates GPI levels in skeletal muscle cells; we treated C2C12 muscle cells with an exercise-mimicking chemical, AICAR. AICAR treatment upregulated GPI expression level in C2C12 cell and its secretomes. To confirm the direct effect of GPI on skeletal muscle cells, we treated C2C12 cells with GPI recombinant protein.

**[Results]:**

We found that GPI improved the viability of C2C12 cells. In the in vivo study, the exercise-treated mice group showed upregulated GPI expression in skeletal muscle. Based on the in vitro study results, we speculated that expression level of GPI in skeletal muscle might be associated with muscle function. We analyzed the association between GPI expression level and the grip strength of the all mice group. The mice group’s grip strengths were upregulated after 2 weeks of treadmill exercise, and GPI expression level positively correlated with the grip strength.

**[Conclusion]:**

These results suggested that the exercise-induced GPI expression in skeletal muscle might have a positive effect on skeletal muscle function.

## INTRODUCTION

Glucose-6-phosphate isomerase (GPI) plays an important role in glycolysis, where it catalyzes the interconversion between glucose-6-phosphate and fructose-6-phosphate^1^. However, it also plays an important role as an angiogenic factor and neurotrophic factor^2,3^. Especially, angiogenesis through GPI is related to hypoxia-induced VEGF upregulation^2^. Secreted GPI can act like a cytokine or growth factor^4^. GPI has several names based on its specific role; these include autocrine motility factor (AMF) and neuroleukin maturation factor. AMF acts like cytokines, and is also known to be a stimulator of vascular proliferation^5^. These signaling pathways are initiated by its binding to specific glycoproteins proteins present on the cell surface^6^, by means of which it acts in an autocrine manner^7^. A previous study showed that GPI mRNA level decreased with aging in gastrocnemius muscle. In addition, they confirmed the upregulation of GPI mRNA in caloric-restricted state^8^. In neural progenitor cells, GPI increases neural differentiation^9^. Furthermore, in fibrosarcoma cells (HT1080), treatment with GPI inhibitor increased oxidative stress, which was confirmed by SA beta-gal staining and GPI knockdown in HT1080 cells. It was reported that knockdown of GPI induces downregulation of aging markers, p21 and p53^10^. 

Secretome is a molecule secreted by cells into the extracellular space of cells or tissues under specific conditions or situations^11^. It is already known that skeletal muscle secretes lots of secretome and acts as an endocrine organ that affects the other organs^12^. In addition, skeletal muscles also secrete autocrinally active molecules, which cause regeneration of muscle itself^13^. Many studies have reported that muscle functions are improved by exercise^14,15^, and substances secreted by muscles can also be altered by exercise^16-18^. It is well known that GPI is secreted from skeletal muscle^9^. However, studies on alteration in GPI expression in mice muscles after exercise and its effects on skeletal muscle are unknown. A previous study showed that AICAR (exercise mimicking molecule that upregulates AMPK signaling pathway) induced AMPK signaling pathway that can mimic the effects of endurance exercise, such as treadmill exercise^19^, and another study showed that AICAR-induced AMPK signal pathway can improve grip strength of muscle damaged mouse model^20^. Therefore, in this study, we performed an *in vitro* analysis to investigate the GPI’s direct effects on skeletal muscle and whether it is upregulated by treatment with AICAR dose-dependently and time-dependently in the skeletal muscle cell. In the in vivo study, 2 weeks of treadmill exercise was performed to assess whether GPI is upregulated by exercise or not and to identify the correlation between muscle function and GPI expression levels in skeletal muscle.

## METHODS

### Animal care

Experimental protocols were approved by the Institutional Animal Care and Use Committee (IACUC) of Seoul National University. The IACUC number is SNU-171229-2-3. Groups of 9-week-old mice were divided according to whether they were subjected to 2 weeks of treadmill exercise (YE, n = 5) or not (YC, n = 5). The study used C57BL/6J mice model. The mice were housed in a controlled environment in 12:12 h light-dark cycle at 22 °C. Animal sacrifice and muscle tissue collection were performed 18 h after last bout of exercise and all muscle tissues were collected after sacrifice. All mice were fed with water and food (Rodent NIH-41 Open Formula Auto, Zeigler Bros Inc., USA) *ad libitum. *

### Exercise Protocol

Before 2 weeks of treadmill exercise, mice were subjected to adaptation exercise, in which the mice were familiarized with the treadmill for 15 min/session at a 0 m/min for 3 min, 5 m/min for 2 min, and 8 m/min for 10 min, and 6 degrees incline once a day for 3 days prior to the experimental day. During the 2 weeks of treadmill exercise, mice allocated to perform treadmill running were subjected to 6 degrees incline, and for warm up, they were subjected to a speed of 0 m/min for 2 min, speeds of 5 m/min, 8 m/min, and 10 m/min for 1 min each, and then, 12 m/min for 30 min during the first week. Then, the speed was increased by 2 m/min every week, and cooled down at 5 m/min for 2 min for 1 session (37 min). Two sessions/day were performed. Between the sessions, a break time of at least 1 h was provided. This exercise protocol was a modified version of the protocol described previously by O'Callaghan and Azimi^21,22^. 

### Grip Strength

The grip strength was measured by using Grip Strength Meter (Bioseb, France). The test was performed in all four limbs of the mice by allowing the animals to grasp a grid plate attached to the force gauge, followed by pulling the animals away from the gauge gently; the trial was performed in triplicates. The highest grip strength was recorded. 

### Western blot

Total proteins were extracted using RIPA buffer (ThermoFisher Scientific, #89900, CA, USA), containing phosphatase inhibitor (Roche, #4906845001, Penzberg, Germany) and protease inhibitor (Roche, #4693159001, Penzberg, Germany), separated by SDS-PAGE, and transferred to NC membranes using Iblot 2 NC mini stacks (Invitrogen, #IB23002, CA, USA). Following primary antibodies were used: anti-Glucose-6-phosphate isomerase (Abcam, #ab66340, MA, USA), and anti-GAPDH (Cell signaling technology, #2118, MA, USA); the antibodies were diluted 1:500~5,000 with TBST (Biosesang, #HT2007, Seongnam, Korea) containing 5% skimmed milk. The signals were detected by Immobilion western chemiluminescent HRP substrate (Millipore, #WBKLS0500, Darmstadt, Germany).

### ELISA analysis

The measurement of secreted GPI levels was performed by enzyme-linked immunosorbent assay (ELISA). Glucose-6-phosphate isomerase ELISA kit (mybiosource, MBS268745, CA, USA) was used for analysis. 

### C2C12 skeletal muscle cell culture, treatment with AICAR and GPI, and cell viability measurement 

Dulbecco’s modified Eagle’s medium (DMEM), (Gibco, 11995-065, CA, USA) and fetal bovine serum (FBS), (Gibco, 16000044, CA, USA) were obtained from Gibco, Fisher Scientific. C2C12 cells (ATCC, #CRL-1772, VA, USA) were cultured in covered 6-well and 96-well plates for 48 h in DMEM supplemented with 10% FBS. Cells were treated with varying concentrations of GPI 24 h in DMEM supplemented with 0.5% FBS. Immediately after GPI treatment, for the measurements of cell viability via NADH dehydrogenase activity, the cells were washed with 1x PBS, and then, treated with DMEM supplemented with 10% cell counting kit 8 assay (Dojindo, CK04-05, MD, USA) (110 μL). The absorbance was measured at 450 nm after 4 h with a microplate reader.

### Statistical Analysis 

Statistical analysis was performed using GraphPad Prism 7 software (GraphPad Software). Results were expressed as mean ± SEM. One-tailed or two-tailed unpaired T-test was performed to examine the difference between the two groups. The level of significance was set at p < 0.05. The correlation coefficient between two groups was analyzed by the Spearman coefficient in SPSS 22.0.

## RESULTS

### Exercise mimetic upregulates GPI secretion from skeletal muscle cell

**Figure 1.**
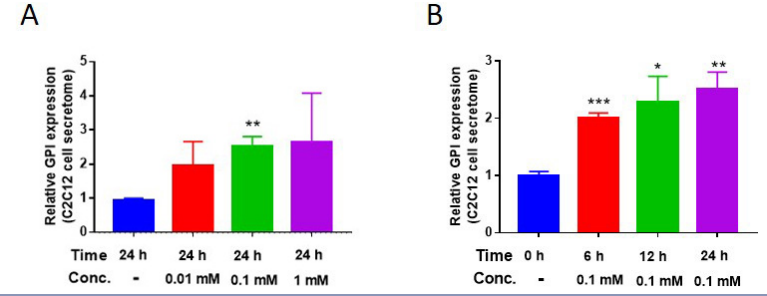
AICAR enhances Glucose-6-phosphate isomerase (GPI) secretion from C2C12 cells. (A and B) Secreted GPI level difference in AICAR-treated C2C12 cell’s secretome, varying in a time- and dose-dependent manner. *p < 0.05, **p < 0.01, ***p < 0.01. All the results were presented as mean ± SEM.

According to a previous study, treatment with AICAR—which is a type of exercise mimetic molecule that upregulates AMPK signaling pathway^19^—upregulated GPI secretion by L6 skeletal muscle cells^9^. However, the result was limited to a single treatment dose and duration. Therefore, we assessed the GPI secretion levels of AICAR-treated C2C12 cells^9^. GPI secretion levels of C2C12 cells were increased after treatment with AICAR in a dose- and time-dependent manner (Fig. 1A and 1B). This result indicated that exercise mimetic could upregulate GPI secretion levels from the skeletal muscle cells. Furthermore, GPI acts in an autocrine manner with its own receptor, known as autocrine motility factor receptor (AMFR)^23,24^, and therefore, secreted GPI from skeletal muscle can act on the skeletal muscle itself directly.

### Exercise mimetic upregulates GPI expression level in skeletal muscle cell

**Figure 2.**
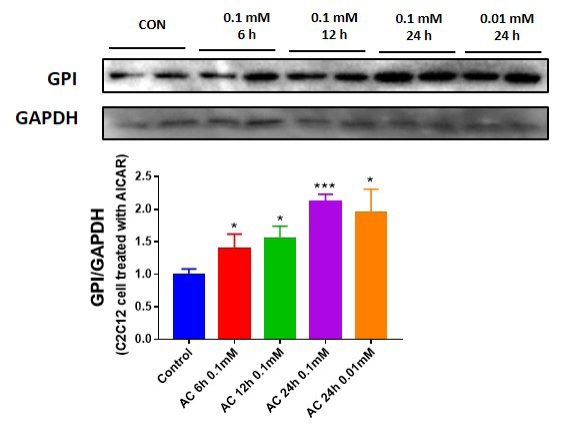
AICAR upregulates GPI expression level in C2C12 cells. (A) Intracellular GPI expression level difference in AICAR-treated C2C12 cell, varying in a time- and dose-dependent manner.

GPI can act in an autocrine manner through its receptor^9^, therefore, we investigated whether exercise mimetic could upregulate GPI expression level in skeletal muscle cells. When C2C12 cells were treated with 5-aminoimidazole-4-carboxamide ribonucleotide (AICAR), GPI expression levels were significantly upregulated in a time- and dose-dependent manner (Fig. 2A). Treatment with an exercise mimetic, such as AICAR, upregulates GPI expression in muscle cell (Fig. 2A) and causes extracellular release of GPI (Fig. 1)

### GPI improves C2C12 cell viability

**Figure 3.**
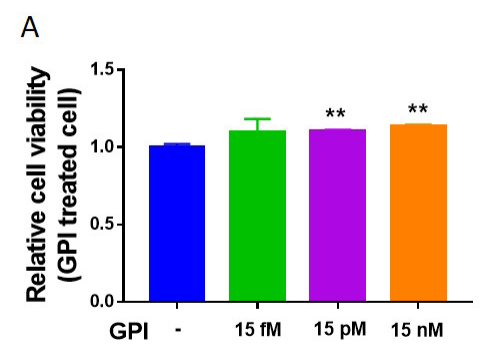
GPI isomerase improves C2C12 cell viability. (A) Relative viability level of C2C12 cells treated with GPI. *p < 0.05, **p < 0.01 vs. GPI negative. All the results were presented as mean ± SEM.

The receptor for GPI is present on skeletal muscle cells. Therefore, GPI signaling pathway could be induced by its direct treatment. To elucidate the direct effects of GPI on the skeletal muscle cells, we treated the C2C12 cells with varying doses of GPI recombinant protein (Enquirebio, #P6112). After treatment with 15 pM and 15 nM of GPI, the viability of C2C12 cells enhanced significantly (Fig. 3A). We measured cell viability using cell counting kit 8 assay (Dojindo, CK04-05), which measures cell viability by evaluating NADH dehydrogenase activity which is related to mitochondrial function^25^. Furthermore, a previous study suggests that mitochondrial activity and muscle function are correlated with each other^26^. This finding suggested that GPI could act directly on skeletal muscle cells and positively influence skeletal muscle function.

### Exercise enhances GPI expression levels that are correlated with muscle function

**Figure 4.**
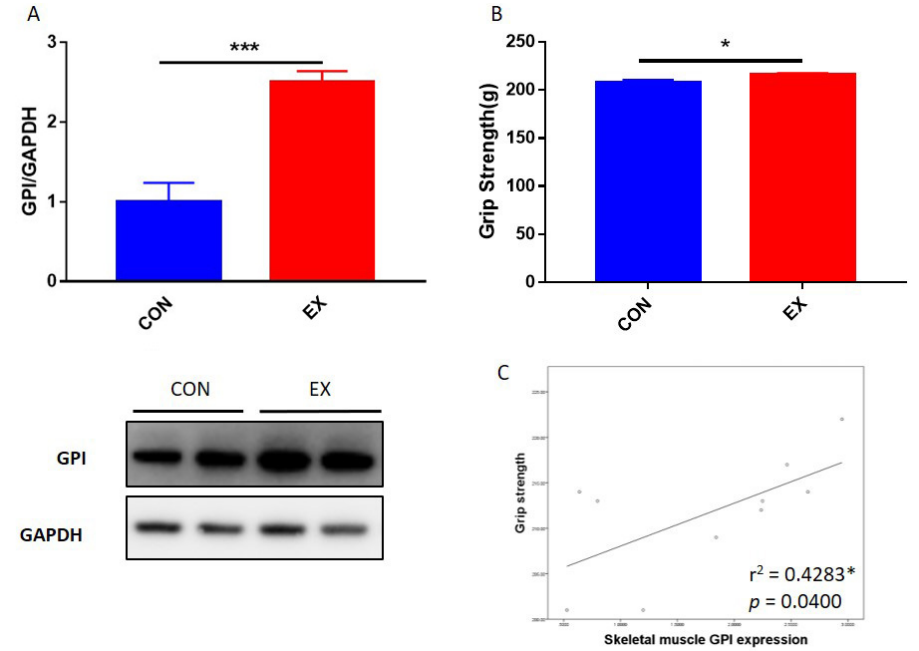
Correlation between muscle function and GPI expression level. (A) GPI expression levels in skeletal muscle of CON (n = 5) and EX (n = 5) groups. (B) Grip strengths of CON and EX mice groups after 2 weeks of treadmill exercise. (C) The correlation of grip strength with GPI expression level in mice skeletal muscle. The lines indicate linear regression, and the Spearman correlation coefficient is shown. *p < 0.05, **p < 0.01, ***p < 0.001.

To confirm whether skeletal muscle GPI expression levels are upregulated by exercise in an animal model or not, we investigated the skeletal muscle GPI expression levels of control mice (CON) and mice subjected to 2 weeks of treadmill exercise (EX). EX group exhibited higher GPI expression level than CON group (Fig 4A). Therefore, these results suggested that GPI expression level in skeletal muscle was upregulated by exercise. In vitro study revealed that GPI upregulated skeletal muscle cell viability, suggesting that GPI might have a positive effect on skeletal muscle. Furthermore, we determined the correlation between skeletal muscle GPI expression level and grip strength of mice groups (CON and EX). Skeletal muscle GPI expression levels showed a positive correlation with the grip strength of the all mice group (Fig 4C). As exercise upregulated grip strength of the mice (Fig 4B), it can be speculated that exercise-induced GPI expression might improve muscle function.

## DISCUSSION

One of the roles of GPI, is catalyzing the interconversion between glucose-6-phosphate and fructose-6-phosphate during glycolysis^2^. Another important role of GPI that was first found three decades ago, was that of a neuroleukin that helps in the survival of skeletal motor and sensory neurons^27^. After that, its other roles as an angiogenic factor^24^, autocrine motility factor^28^, and its effect on rheumatoid arthritis^29^ have been revealed. However, even though the human protein ATLAS upregulated GPI expression in skeletal muscle, there has been little research on the relationship between GPI and skeletal muscle. Thus, in this study, we aimed to determine the relationship between GPI and skeletal muscle function. 

In our experiments, the viability of GPI-treated muscle cells was investigated. GPI treatment enhanced the muscle cell viability in a dose-dependent manner (Fig. 3A). Previous studies have shown that when GPI is knocked down in lung fibrosarcoma cells, the levels of cell senescence-related molecules such as p53 and ROS increased^10^ and the number of cells decreased^30^. Another study showed that p53 overexpression induced muscle atrophy, and p53 downregulation delayed immobilization-induced atrophy^31^. In addition, upregulation of ROS induced atrophy of skeletal muscles^32^. Therefore, it can be suggested that GPI upregulates skeletal muscle cell viability via inhibition of p53 and ROS signaling pathways. The results of these studies seem to be consistent with the results obtained after GPI treatment on muscle cells. However, in this study, when GPI was directly applied to skeletal muscle cells, the markers related to the increase and the loss of muscle were not confirmed in this study. Therefore, it is important to confirm the markers related to the increase and the loss of skeletal muscle in future studies.

Cheol-Koo Lee’s study in 1999 aimed to analyze the effects of aging and caloric restriction on mouse skeletal muscle mRNA expression^8^. In a previous study, Cheol-Koo Lee validated increase in skeletal muscle GPI expression following caloric restriction. Other studies have shown that caloric restriction and exercise increase the AMP/ATP ratio and promote the AMPK signaling pathway. Therefore it can be speculated that caloric restriction and exercise mediate their effects via the same AMPK pathway^33^. Caloric restriction induces protein breakdown^8^; however, AMPK signaling pathway can delay apoptosis and senescence of cell^34^, and therefore, it could attenuate severe muscle loss^35^. Based on these results, we hypothesized that upregulation of AMPK signaling pathway by exercise and AICAR, which can stimulate AMPK in muscle cells to mimic exercise, could increase GPI expression level in skeletal muscle. Intracellular GPI expression level was upregulated in a time- and dose-dependent manner following by AICAR treatment (Fig. 2A). In addition, the secretion levels of GPI was also increased after AICAR treatment in a concentration- and time-dependent manner (Fig. 1A, 1B). We also found that secreted GPI level was upregulated in AICAR-treated L6 cells, as shown in a previous study^9^. 

It is well known that exercise improves muscle function^36,37^, and several previous studies have suggested that exercise-induced substances improved muscle function^13,38^. Therefore, in this study, we determined the enhancement of muscle function and GPI expression following exercise. Then, based on the results of the *in vitro* experiments, it was speculated that GPI expression affects the skeletal muscle function. Further, a correlation between the muscle function and the GPI expression in skeletal muscles of mice was established (Fig. 4A, 4B, and 4C). We found that the exercise-induced expression of GPI was positively correlated with skeletal muscle function. However, this study did not reveal the mechanism of GPI action on the animal skeletal muscle; this can be elucidated in future studies.

## CONCLUSION

GPI is found abundantly in cells because it is involved in the glycolysis. Through this study, it was confirmed that exercise can increase GPI expression in muscles. In addition, it was confirmed that GPI expression is positively correlated with skeletal muscle function. Therefore, further studies will be needed to elucidate the effect of exercise-induced GPI on skeletal muscle function.
